# Editorial: Melanocortins and melanocortin receptors in the regulation of inflammation: mechanisms and novel therapeutic strategies

**DOI:** 10.3389/fimmu.2023.1226886

**Published:** 2023-05-31

**Authors:** Petteri Rinne, Andrew W. Taylor, Trinidad Montero-Melendez

**Affiliations:** ^1^Research Centre for Integrative Physiology & Pharmacology, Institute of Biomedicine, University of Turku, Turku, Finland; ^2^Turku Center for Disease Modeling, University of Turku, Turku, Finland; ^3^Chobanian & Avedisian School of Medicine, Boston University, Boston, MA, United States; ^4^The William Harvey Research Institute, Queen Mary University of London, London, United Kingdom

**Keywords:** melanocortin (MC), melanocortin 1 receptor, inflammation, colitis, arthritis (including rheumatoid arthritis), fibroblast, drug development, Alzheimer’s disease

Melanocortins (MC) are derived from post-translational processing of the pro-opiomelanocortin (POMC) precursor protein and consist of melanocyte-stimulating hormones (α-, β- and γ-MSH) and adrenocorticotropic hormone (ACTH). These peptide hormones regulate important physiological functions, including skin pigmentation, steroidogenesis, and inflammation, by activating a family of G-protein coupled melanocortin receptors (named from MC_1_ to MC_5_). Over the past decades, basic and translational research has firmly established that melanocortins favorably modulate both innate and adaptive immune responses and provide therapeutic benefits in various experimental models of acute and chronic inflammation. These findings have also sparked interest within the pharmaceutical industry to develop MCR targeted compounds as novel anti-inflammatory therapies for clinical conditions like inflammatory bowel disease and rheumatoid arthritis.

Due to the short half-life of α-MSH, synthetic analogs of this naturally occurring peptide have been developed and characterized, one of them being NDP-α-MSH (also known as afamelanotide or melanotan-1) (1). It is widely used as a pharmacological tool in melanocortin research with high potency at MCRs and improved pharmacokinetic profile. It is also the active compound of Scenesse^®^ implant that has been approved for treating erythropoietic protoporphyria and actively investigated as a treatment for other skin diseases. Daini et al. used NDP-α-MSH as a pan-agonist of MC receptors and expanded its potential therapeutic effects into experimental Alzheimer’s disease (AD). Although the neuroprotective effects of this compound have been proven in models of early AD (2, 3), Daini E et al. demonstrated that NDP-α-MSH improved cognitive function in two different transgenic mouse models that manifested more advanced stages of AD. The improvement in cognitive abilities was associated with modest changes in the pathological hallmarks of AD, including reduced microglial activation. The results strengthen the earlier findings and implicate the neuroprotective effects of NDP-α-MSH treatment. Still, the underlying mechanisms regarding the involved MCR subtype(s) and effector cells remain largely unclear.

Other synthetic analogs structurally resembling endogenous α-MSH peptide have been recently developed and are also being investigated in clinical trials. One of them is PL8177 (Palatin Technologies, Inc, Cranbury, NJ, USA), which selectively activates mouse and human MC_1_ and has been shown to protect against intestinal and ocular inflammation in preclinical disease models with efficacy similar to α-MSH (4). Following the finding that intracolonic administration of PL8177 was effective in a rat model of inflammatory bowel disease (4), an oral formulation of PL8177 was developed and investigated for its therapeutic value in experimental colitis models. Dodd et al. demonstrated that this oral formulation significantly reduced colon damage scores and prevented the disruption of colon structure and barrier in rats with chemically induced colitis. Paving the way for further clinical investigation, pharmacokinetic studies conducted in animals and humans showed that PL8177 was released from the orally administered polymer formulation, leading to high concentrations of the compound in the colon. At the same time, PL8177 and its main metabolite were undetectable in the plasma and urine.

Garrido-Mesa et al. investigated the anti-inflammatory potential of PL8177 *in vitro* and in a mouse model of inflammatory arthritis. They demonstrated that it induces cAMP production and ERK1/2 phosphorylation as intracellular signaling messengers in HEK293A cells transiently transfected with mouse or human MC_1_. PL8177 showed strong selectivity for MC_1_ and even higher potency (*i.e.*, lower EC_50_ values) in cAMP and ERK1/2 phosphorylation assays compared to α-MSH. In *in vitro* assays with primary mouse macrophages, PL8177 downregulated pro-inflammatory genes such as *Il1b* and *Il6* and promoted macrophage efferocytosis, indicating pro-resolving properties of the compound. In a mouse model of arthritis, PL8177 dose-dependently attenuated signs of inflammation and swelling in the arthritic joints, which was accompanied by a reduction in leukocyte infiltration, particularly in infiltrating neutrophils and monocytes. These findings further consolidate the therapeutic potential of targeting MC_1_ for inflammatory diseases and extend the potential clinical utility of PL8177 or other similar MC_1_ selective agonists to inflammatory arthritis.

The presence of MC_1_ in different leukocyte subpopulations, such as monocytes and macrophages, is an intriguing starting point for developing novel MC_1_-targeted therapies. However, expression of *MC1R* has also been reported in fibroblasts (5, 6), which are the workhorse for producing structural proteins of extracellular matrix (ECM) and important regulators of immune responses. Khodeneva et al. elegantly review the regulatory role of fibroblasts in health and disease and discuss the potential of targeting MC receptors to treat diseases driven by aberrantly activated fibroblasts. *In vitro* studies have proven that α-MSH and selective MC_1_ agonists such as BMS-470539 reduce fibroblast activation, proliferation, ECM deposition, and inflammatory markers. Regarding the involved intracellular signaling pathway, it appears that the effects are mediated by ERK1/2 phosphorylation instead of the classical Gs/cAMP/PKA pathway. This opens a possibility for pathway-selective drug targeting by biased agonism, an emerging concept in the melanocortin-related drug development (7, 8). Therapeutically, the antifibrotic effects mediated by α-MSH and MC_1_ activation are of high relevance in a variety of diseases such as skin and lung fibrosis, as already evidenced by preclinical studies (9–11). Indeed, novel MCR agonists such as AP1189 and MT-7117 (dersimelagon) are currently being investigated in phase 2 clinical trials for efficacy and safety in subjects with rheumatoid arthritis and systemic sclerosis, respectively (8).

In conclusion, the role of melanocortins and their cognate receptors in modulating immune responses has been known for some time, but only recently has evidence unequivocally indicated their therapeutic potential in various inflammatory and fibrotic diseases, as exemplified by the ongoing clinical trials evaluating the safety and efficacy of novel MCR targeting drugs. This Research Topic provides new insight into the therapeutic landscape related to the ‘old and new’ melanocortin drugs.

## Author contributions

PR, AT, and TM-M wrote the editorial and invited authors to contribute to the Research Topic. All authors contributed to the article and approved the submitted version.
